# Functional lung morphometry: another piece in the BPD prediction puzzle?

**DOI:** 10.1038/s41390-023-02701-3

**Published:** 2023-06-23

**Authors:** Qi Hui Poh, Sophia Dahm, David G. Tingay, Arun Sett

**Affiliations:** 1 Neonatal Research, Murdoch Children’s Research Institute, Parkville, VIC Australia; 2 Department of Paediatrics, University of Melbourne, Melbourne, VIC Australia; 3 Newborn Services, Joan Kirner Women’s and Children’s, Sunshine Hospital, Western Health, St Albans, VIC Australia; 4 Department of Obstetrics and Gynaecology, University of Melbourne, Melbourne, VIC Australia

Despite considerable advancements in neonatal respiratory care, bronchopulmonary dysplasia (BPD) remains a burden for many survivors of premature birth.^1^ Rates of BPD remain high and currently, clinicians have few therapies which effectively reduce BPD. Compounding this, many necessary treatments in the neonatal intensive care unit (NICU) can increase the risk of acute lung injury, and thus BPD. Identifying specific interventions that are most likely to be beneficial, or harmful, involves understanding the risk profile of specific infants. To do so requires clinically quantifiable measures which may assist in better characterising the target population. As our understanding of BPD and the profile of preterm infants managed in the NICU has changed since the first description of BPD in 1969, so has the focus on relevant biomarkers which may predict a diagnosis of BPD early in life.^2^

In this issue of *Pediatric Research*, Williams et al. report a novel approach to potentially predict BPD in preterm infants using functional respiratory system morphology that calculates an index of the adjusted alveolar surface area of the lung (S_A_).^3^ The aim of their observational prospective study was to describe adjusted S_A_ values in preterm infants at high risk of BPD and explore whether adjusted S_A_ was related to respiratory outcomes of prematurity known to be associated with BPD. Thirty extremely premature infants born <28 completed weeks’ gestation who were still receiving invasive mechanical ventilation at 7 days were recruited for this study. They identified a lower adjusted S_A_ between those infants who were discharged home on oxygen and those that were not (a functional surrogate of BPD at discharge); median (range) 637 (232–838) cm^3^ versus 799 (444–903) cm^3^. There was no relationship between adjusted S_A_ and grade of BPD or duration of mechanical ventilation. There is a strong biological rationale to use S_A_ to describe respiratory status in preterm infants. Preterm birth requires tidal ventilation of an immature lung before alveolarisation has become the predominant developmental process, and often whilst in the saccular or canicular stages of lung development. Beyond these immediate consequences, mechanical ventilation causes alveolar developmental arrest leading to a smaller alveolar surface area, simplified alveolar structure and airway enlargement (increase in deadspace). The physiological manifestation of the altered capacity of alveolar-capillary gas exchange, and potential for supplementary oxygen, is thus related to lung morphology.

It is important to focus on the sophisticated process used to determine the adjusted S_A_ index by Williams et al. First, the alveolar ventilation/perfusion ratio (V_A_/Q) of each infant was calculated from at least three paired measurements of transcutaneous oxygen saturation (SpO_2_) and fraction of inspired oxygen (FiO_2_) whilst supine. The V_A_/Q was then considered within the expected oxyhaemoglobin dissociation reference curve and adjusted for haemoglobin. From the V_A_/Q, the alveolar surface area of the lung (S_A_) was estimated using a predictive regression equation from a previous study which validated functional morphometry with traditional stereological morphometry in a preterm baboon model. To further ensure the transferability of this regression equation to human data, and reduce confounding variables for V_A_/Q, echocardiography was used to ensure left-to-right ductal flow and similar pulmonary perfusion and alveolar-arterial gradient between the baboon model and human data. The resultant S_A_ was finally adjusted by accommodating anatomical and physiological alveolar deadspace as well as alveolar tidal volume (V_T_) measured by mainstream volumetric capnography. These adjustments were used to develop a more precise index of the functional S_A_ taking part in gas exchange. In ideal measuring conditions, capnography allows differentiation of the alveolar and deadspace volumes. The need to correct for estimated deadspace accounts for the unique lung architecture in preterm infants with evolving chronic lung changes, which potentially include a substantial proportion of underdeveloped or damaged alveoli. The authors assessed the predictive capabilities of both S_A_ and adjusted S_A_ index to measure clinically relevant indicators of BPD (grade, need for home oxygen and duration of mechanical ventilation).

The novelty and strength of this study is the generation of a repeatable estimation of total S_A_ from a careful translation of a methodology previously validated against stereological morphometry in post-mortem preterm baboon lung tissue.^4^ The method of S_A_ estimation used by Williams et al. has the advantage of using physiological measurements readily obtained from commonly available NICU equipment without the need for interruption of clinical care (such as inert gas washout). Furthermore, the computer-based calculation used has substantial potential for advancement, including speed, automation, and high throughput, which reduces the risk of human error and variability to produce consistent results. This allows immediate information on disease state or treatments. The adjusted S_A_ index could also be applied to other respiratory diseases of infancy. The range of adjusted S_A_, deadspace and V_A_/Q values suggest that identifying a single narrow range of reliable, informative, and accurate absolute values is unlikely. Rather, repeated measures and intra-subject comparison of the evolution of lung dysfunction informed by associated clinical events will be more useful.

Current diagnostic criteria for BPD reflect a simplistic snapshot in time. Predicting BPD risk is thus appealing, but the search for the best biomarkers for the prediction of BPD has been challenging.^5^ This is because BPD results from complex interactions between the developmental lung state, treatments and complications, all of which influence the trajectory to BPD and may occur after an early tool has estimated risk. Clinical, physiological, biochemical and, most recently, imaging with lung ultrasound (LUS) have been proposed as biomarkers, each with distinct advantages and disadvantages. Biochemical markers (generally umbilical cord blood, plasma, urinary and tracheal aspirates) have shown promise and directly address the multi-modal inflammatory nature of BPD, but have the disadvantage of invasiveness and off-site sample processing.^5–7^ Recently, the predictive performance of LUS has been promising in some, but not all, studies.^8, 9^ However, LUS is an acquired skill which requires training. In contrast to these measures, adjusted S_A_ is a method without these limitations, nor limited by age and vulnerable patient cohorts. Although the adjusted S_A_ in the second week after birth did not significantly correlate with the duration of supplemental oxygen therapy or grade of BPD, it did predict discharge with supplemental oxygen with reasonable accuracy (AUC 0.815, sensitivity; 86%, specificity; 77%), both statistically and against other early BPD predictors. It should be noted that the study did not use adjusted S_A_ to predict BPD, as only a population of infants in whom the investigators determined would develop BPD were studied. It is unclear if this test would have the same predictive performance in the general preterm population for the outcomes studied, and BPD generally.

A surprising finding of the study was the high physiological and anatomical deadspace measures, median (range) 5.8 (3.9–9.7) and 5.1 (3.6–7.4) mL/kg, respectively, and an alveolar V_T_ below that considered appropriate for gas exchange (1.90 (0.54–6.33) mL/kg). The authors propose that this may reflect the under-appreciated and novel mechanisms of gas exchange (such as fresh gas spikes through deadspace). The true answer is probably more complex. Volumetric capnography assesses the behaviour of CO_2_ over the duration of the expiratory cycle, with deadspace gas representing the initial CO_2_ changes and alveolar gas only being present at the end of expiration (CO_2_ wave plateau). Although the authors used a low-volume mainstream capnograph, the low V_T_ and fast respiratory rates characteristic of preterm respiration can result in over-representation of deadspace.^10^ The study population had all received prolonged mechanical ventilation beyond the initial period of acute respiratory distress syndrome. It is likely that airway and alveolar injury and changes had already occurred. Finally, pendelluft gas flow (movement of air within the lung) during tidal inflations may be common in adults with acute respiratory disease syndrome receiving mechanical ventilation. Whether this also occurs in this population of high-risk preterm infants is a fascinating proposition.

Minimally invasive estimation of S_A_ appears suitable for use in extremely preterm neonates, and may provide important information on gas exchange, lung function and disease pathophysiology not currently available in the NICU. This opens exciting avenues for precision medicine to target specific hallmarks or stages of disease. Given the chronic and detrimental effects of BPD-associated damage on lung development and function in later life, the potential of adjusted S_A_ as an early prognosticator of later respiratory risk is appealing. However, standardisation of the method (for example against ventilation support settings), inclusion of a lower-risk population including those on non-invasive ventilation, and temporal tracking of S_A_ to monitor disease progression should be conducted to demonstrate robustness and reproducibility. Even with these data, it is unlikely that accurate prediction of BPD or its clinical manifestations can be based on S_A_ measurements alone. The multi-modal nature of evolving chronic lung disease following preterm birth indicates that accurate diagnosis and prediction of BPD will require multiple validated tools encompassing physiological measures, imaging modalities, markers of immunomodulatory, inflammatory, and oxidative stress, ventilatory treatments and clinical factors (such as degree of prematurity and infection). The work of Williams et al suggests that adjusted S_A_ may play an important part in this endeavour.
